# Study on the Influence Mechanism of Mineral Admixtures on Hydration and Microstructure of Yellow River Sediment-Based Shotcrete

**DOI:** 10.3390/ma19122532

**Published:** 2026-06-11

**Authors:** Ge Zhang, Chen Chen, Zekun Dong, Jialing Li, Kunpeng Li, Ali Raza, Chengfang Yuan

**Affiliations:** 1 Yellow River Institute of Hydraulic Research, Yellow River Water Conservancy Commission, Zhengzhou 450003, China; 2 Key Laboratory of the Yellow River, Ministry of Water Resources, Zhengzhou 450003, China; 3 School of Water Conservancy and Transportation, Zhengzhou University, Zhengzhou 450001, China; 4 School of Civil Engineering, Zhengzhou University, Zhengzhou 450001, China

**Keywords:** Yellow River Sediment (YRS), shotcrete, mineral admixtures, characteristic products, microstructure

## Abstract

This study investigates the effects and mechanisms of three mineral admixtures—fly ash (FA), silica fume (SF), and metakaolin (MK)—on the fresh, mechanical, and microstructural properties of Yellow River sediment (YRS)-based shotcrete. A comprehensive experimental program was conducted, including setting time determination, workability assessment, and mechanical strength evaluation, complemented by microstructural characterization using X-ray diffraction (XRD), Fourier transform infrared spectroscopy (FT-IR), thermogravimetric analysis (TGA), and scanning electron microscopy (SEM). The results indicate that the incorporation of FA prolonged initial and final setting times and improved pumpability but reduced build-up thickness and compressive strength; splitting tensile strength at later ages remained comparable to the control. SF shortened the final setting time and reduced flowability but enhanced shootability, layer build-up, and medium- to later-age compressive and tensile strengths, with an optimal dosage of 5%. MK accelerated the final setting time, slightly reduced early-age compressive strength, but improved early-age splitting tensile strength and achieved 28-day compressive strength comparable to the control. Microstructural analyses revealed that FA participates in pozzolanic reactions forming C–(A)–S–H gel, while SF and MK promote the formation of dense C–S–H and carboalumination phases, enhancing matrix densification. Based on performance evaluation, the recommended dosages are FA ≤ 20%, SF ≤ 15%, and MK ≤ 15%. These results establish clear links between macroscopic performance and microstructural evolution, providing experimental guidance for the sustainable development of YRS-based shotcrete.

## 1. Introduction

Shotcrete is a type of concrete that is conveyed through a pneumatic hose or pipe and projected at high velocity onto a receiving surface, being compacted instantaneously under the action of compressed air [1,2]. Due to its rapid setting behavior, fast hydration-induced hardening, high early mechanical performance, and suitability for mechanized construction [3,4], shotcrete has become a widely used construction material in railway, highway, hydraulic, and mining engineering applications [5,6]. However, the large-scale application of shotcrete in infrastructure construction results in a substantial demand for high-quality cementitious materials and natural aggregates, particularly fine aggregates [7,8]. With the progressive depletion of natural river sand resources and the increasing environmental constraints associated with aggregate extraction [9], the development of sustainable alternative materials for shotcrete has become a critical research priority in civil engineering [10]. In this context, the valorization of locally available sediment resources offers a promising pathway for achieving both resource efficiency and environmental sustainability [11,12]. Sustainable regulation and utilization of the Yellow River system are essential for supporting China’s long-term infrastructure development and resource security [13]. However, the basin is increasingly affected by severe sedimentation, which is mainly associated with reduced flow capacity, elevated sediment concentration, and the mismatch between hydraulic transport and sediment load [14,15]. This condition has led to persistent engineering challenges, particularly in terms of flood management efficiency, reservoir sedimentation control, and the durability of irrigation facilities [16,17]. In parallel, the rapid growth of the construction industry has resulted in an exceptionally high demand for granular materials, with annual consumption of sand and gravel approaching 20 billion tons in China [18,19]. The overexploitation of natural sand sources has raised serious sustainability concerns, prompting the need to identify alternative fine aggregates [20]. Within this framework, Yellow River sediment (YRS), characterized by a high silica (SiO_2_) content, can be considered a viable material for partial or full replacement of conventional sand in cement-based composites [21]. Leveraging these sediment resources not only presents significant socio-economic benefits but also contributes to ecological sustainability [22,23].

**Figure 1:**
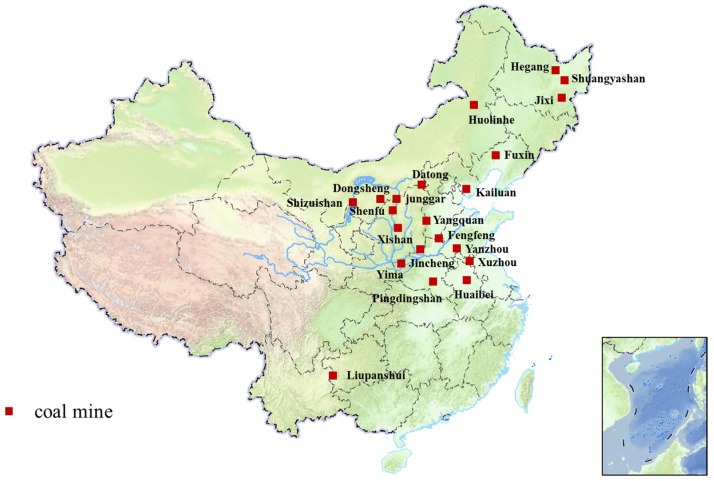
Distribution map of major coal mines in China [29].

The Yellow River Basin is widely recognized as a key energy and industrial hub in China, playing a crucial role in sustaining national energy supply and large-scale manufacturing activities [21]. The region represents a key national coal-resource concentration zone, as nine of China’s fourteen major coal production bases are distributed within the basin, as shown in Figure 1. In addition, provinces such as Shanxi, Henan, and Shandong are dominated by heavy industries, including steelmaking and aluminum production [24,25]. These industrial clusters have significantly accelerated regional economic growth, contributing to rapid industrial expansion and urban development along the Yellow River corridor [26]. Despite these benefits, intensive industrial operations have introduced considerable environmental challenges. In particular, the large-scale generation of industrial by-products, such as fly ash and metallurgical slag, has become a critical issue. Persistent accumulation of these industrial solid wastes has resulted in extensive land occupation and multi-media environmental pollution, including degradation of soil quality, contamination of water resources, and atmospheric emissions, which collectively threaten regional ecological stability [27,28].

Compared with conventional river sand, Yellow River sediment (YRS) exhibits markedly different granulometric and physicochemical properties [30,31]. The material is primarily composed of fine particles, predominantly within the silt and clay size fractions (<0.075 mm), with irregular, flaky, or angular morphologies. In addition, YRS is characterized by a rough surface texture, high specific surface area, and elevated clay mineral content [32]. These intrinsic properties significantly influence its behavior when used as a fine aggregate in shotcrete, resulting in performance characteristics that differ substantially from those of mixtures prepared with natural sand [33,34]. Accordingly, existing findings are not directly applicable to performance prediction for Yellow River sediment-based shotcrete. It is therefore necessary to develop a targeted parameter optimization framework that reflects the specific material characteristics of Yellow River sediment. In recent years, several studies have explored the feasibility of incorporating YRS in cementitious composites, particularly in conventional concrete and engineered cementitious composites (ECC). Chen et al. [35] reported that the gradation design of Yellow River sand in UHPC was refined using the closest packing theory, yielding a 28-day compressive strength of 130 MPa. This demonstrates the potential of YRS to produce high-strength, cost-efficient cement-based materials while promoting the utilization of river sediment. Tan et al. [36] demonstrated that YRS-based ECC can effectively enhance the interfacial bonding performance of frost-damaged concrete, with bond strength increasing by 27–44% under freeze–thaw cycles due to the formation of a denser interfacial transition zone (ITZ). Furthermore, Raza et al. [15] reported that freeze–thaw resistance tests on YRS-based ECC showed that the optimized mix retained 98.5% mass and 77.4% residual flexural strength after 300 cycles, indicating that Yellow River sand can serve as a mechanically stable and durable fine aggregate for ECC applications. Wang et al. [37] prepared lightweight ceramsite using Yellow River sediment and confirmed its suitability as an alternative aggregate in masonry mortar. Complete replacement of natural sand maintained acceptable mechanical performance, indicating the potential for large-scale utilization of YRS in concrete. Despite these advancements, existing studies have predominantly focused on conventionally cast cementitious systems, whereas research on YRS-based shotcrete remains limited and lacks a systematic framework. In particular, the influence of mineral admixtures on the fresh-state behavior, hydration process, and microstructural evolution of shotcrete has not been comprehensively investigated, especially with respect to the synergistic effects of multiple admixtures. This knowledge gap restricts the development of optimized mix designs and limits the practical engineering application of YRS-based shotcrete. Based on the above considerations, this study employs fly ash (FA), silica fume (SF), and metakaolin (MK) as representative mineral admixtures to partially replace cement. A series of mixtures with varying replacement ratios was designed to systematically evaluate their effects on setting time, workability (including pumpability and spray ability), hydration process, and mechanical properties of shotcrete. Furthermore, TG–DTA, XRD and SEM–EDS analyses were conducted to examine the influence of mineral admixtures on hydration products, pore structure, and matrix microstructure. The objective of this study is to develop a systematic correlation between engineering performance, microstructural development, and intrinsic mechanisms, with the aim of providing theoretical support and technical references for the sustainable utilization and field application of YRS shotcrete incorporating multi-source solid wastes.

## 2. Materials and Methods

### 2.1. Constituent Materials and Mix Design

#### 2.1.1. Raw Material

**Figure 2:**
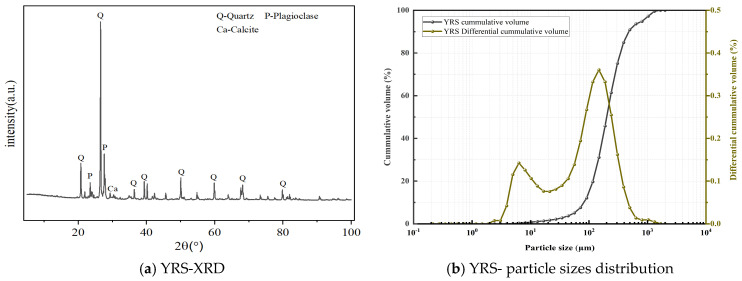
Physical and chemical properties of YRS.

**Figure 3:**
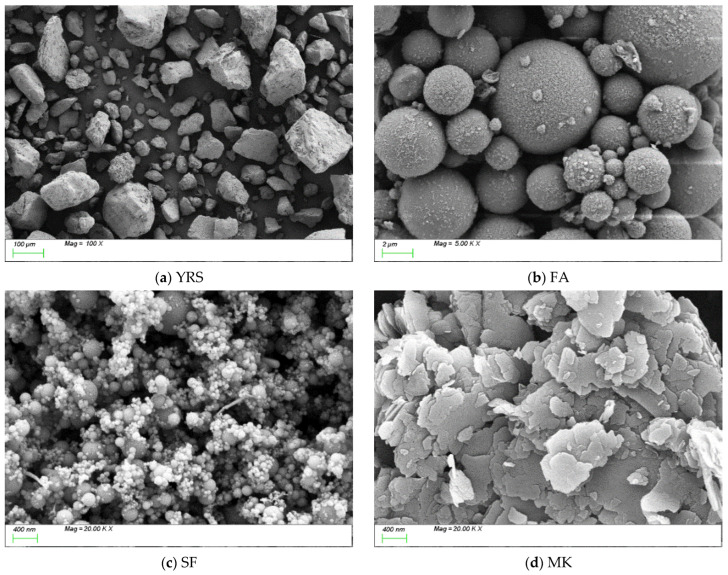
SEM images of YRS and mineral admixtures [29].

The materials used in this study comprised ordinary Portland cement, Yellow River sediment (YRS), fly ash (FA), silica fume (SF), metakaolin (MK), and an alkali-free setting accelerator. The Yellow River sediment (YRS) was obtained from the Xixiayuan Reservoir in Henan Province, China. The collected material was in a wet condition and was later oven-dried before use to achieve uniform moisture content and ensure experimental consistency. The main mineral compositions in the sediment include quartz, plagioclase, and calcium carbonate (Figure 2a). The particle sizes of the YRS were analyzed using a Malvern Mastersizer 2000 laser particle-size analyzer (Malvern Panalytical, Worcestershire, UK). The results are presented in Figure 2b. Ordinary Portland cement (P·I 42.5), conforming to the Chinese National Standard GB 8076 [38] (equivalent to CEM I 42.5), was used as the primary binder. SF used in this study had a density of 2.214 g/cm^3^, a very high specific surface area of 18,000–20,000 m^2^/kg, and a SiO_2_ content exceeding 92%, with an average particle size of 16.88 μm. MK was also incorporated, exhibiting 7-day and 28-day activity indices of 116% and 119%, respectively. Grade I fly ash, with a density of 2.18 g/cm^3^, was incorporated as a supplementary cementitious material. The results are presented in Table 1. Microstructural morphology was examined using scanning electron microscopy (SEM), as shown in Figure 3. The YRS particles exhibited irregular geometries with significant size variability, which is expected to influence packing density and interparticle friction. The mineral admixtures displayed distinct morphological characteristics: MK appeared predominantly as flaky agglomerates, whereas FA and SF consisted mainly of spherical particles, contributing to improved particle packing and potential ball-bearing effects. An alkali-free liquid accelerator (SBT^®^-N (II) type, Sobute New Materials Co., Ltd., Nanjing, China) was used to regulate the setting behavior and early-age strength development of the shotcrete mixtures.

#### 2.1.2. Mix Proportion

In this study, the effects of mineral admixtures on the hydration characteristics and microstructural evolution of Yellow River sediment (YRS)-based shotcrete were systematically investigated through a series of setting time tests, fresh-state performance evaluations (workability), mechanical strength measurements, and microstructural characterization. Table 2, Table 3 and Table 4 summarize the mixture proportions adopted for the setting time, workability, and mechanical and microstructural tests, respectively. To ensure consistency and facilitate comparison among different mixtures, all numerical values presented in these tables were normalized prior to analysis. The setting time tests were conducted using paste mixtures with FA, SF, and MK replacement levels of 10%, 20%, and 30%, respectively, in order to preliminarily evaluate the influence of a relatively wide dosage range on the early setting behavior of the binder system. In contrast, the shotcrete mixtures were designed based on practical workability and mechanical performance considerations. Therefore, FA was incorporated at 10%, 15%, and 20%, while SF and MK were incorporated at 5%, 10%, and 15%. The lower upper limit for SF and MK was selected because these materials have higher fineness and reactivity, and excessive contents may significantly increase water demand, reduce workability, and affect the spraying performance of shotcrete. The mixture design was developed to evaluate the influence of different types and replacement levels of mineral admixtures on the performance of YRS-based shotcrete under consistent mix conditions.

### 2.2. Experimental Methodology

This study systematically evaluates how fly ash (FA), silica fume (SF), and metakaolin (MK) affect the fresh-state properties, mechanical response, hydration evolution, and microstructural development of shotcrete. The experimental program included setting time determination, workability evaluation (pumpability and shootability), mechanical strength testing, and microstructural characterization. The latter comprised thermogravimetric analysis (TGA), X-ray diffraction (XRD), Fourier transform infrared spectroscopy (FT-IR) and scanning electron microscopy (SEM). Table 5 summarizes the experimental program, including the performance indices and specimen dimensions.

#### 2.2.1. Setting Time Test

The setting characteristics were determined following the Chinese standard GB/T 35159 [39] for shotcrete accelerators. All paste mixtures were prepared with a fixed water-to-binder ratio of 0.35. To ensure reliability, each mixture was tested in triplicate, and the mean value was taken as the representative result.

#### 2.2.2. Workability Test

The fresh-state performance was assessed through pumpability and shootability indicators. Pumpability was quantified using slump and slump flow tests, which reflect flow resistance and transport efficiency of the mixture. The workability of the fresh mixtures was evaluated by slump and slump flow tests, which were conducted in accordance with ASTM C143/C143M and ASTM C1611/C1611M, respectively. Shootability was evaluated in terms of rebound ratio and single-layer build-up thickness, representing material loss during spraying and the stability of deposited layers.

#### 2.2.3. Mechanical Property Testing

To comprehensively investigate the influence of fiber reinforcement on strength and toughness, 100 mm cube specimens were cast for compressive and splitting tensile strength tests. After demoulding, the specimens were cured in a standard environment (20 ± 2 °C, relative humidity > 95%) until the designated testing age, in accordance with GB/T 50081-2019 [40]. The tensile–compressive strength ratio was subsequently calculated based on the obtained results.

#### 2.2.4. Infrared Spectroscopy Analysis

Phase composition of surface products was examined using an FTIR spectrometer (XploRA PLUS, Horiba, France). Spectral data were recorded at room temperature using a 785 nm laser source over the wavenumber range of 100–4000 cm^−1^. Measurements were performed with a 50× objective lens, providing a laser spot size of approximately 1 μm and an output power of 1 mW.

#### 2.2.5. X-Ray Diffraction Analysis

Phase identification was conducted using an X-ray diffractometer. Prior to testing, samples were oven-dried, ground into fine powder, and placed in a glass sample holder. Scanning was performed over a 2θ range of 5–70°, with a step size of 0.04° and a scanning rate of 2°/min.

#### 2.2.6. Thermogravimetric Analysis

Thermal behavior of the samples was evaluated. Approximately 15 mg of powdered material was used for each test. The specimens were heated at a constant rate of 10 °C/min up to a maximum temperature of 1000 °C. Differential thermal analysis (DTA) curves were recorded to characterize phase transformations and decomposition behavior.

#### 2.2.7. Scanning Electron Microscopy Test

The microstructure of the specimens was characterized using a Sigma 300 scanning electron microscope (Carl Zeiss, Oberkochen, Germany). Specimens cured for 28 days were fractured to expose fresh surfaces, followed by hydration termination prior to testing to preserve the internal structure [41].

## 3. Experiment Results and Analysis

### 3.1. Setting Time

**Figure 4:**
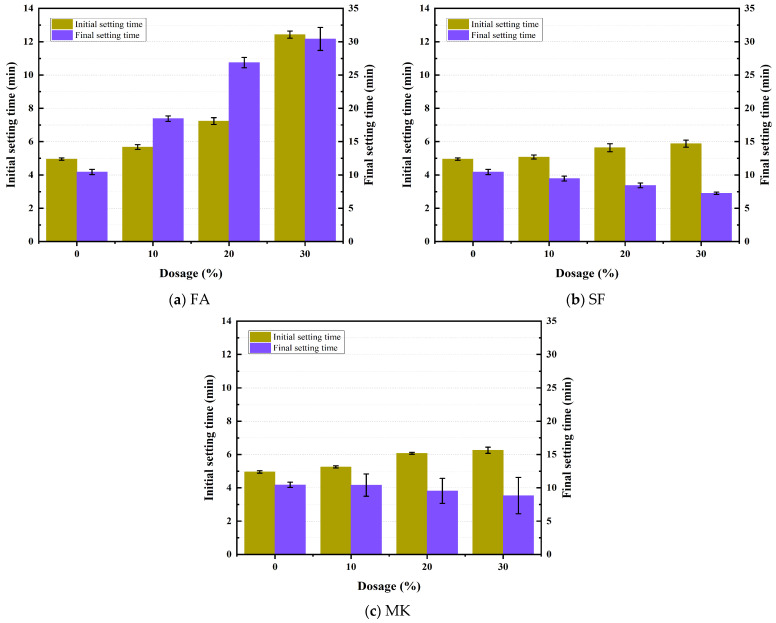
Effect of mineral admixtures on the setting time of Yellow River sediment shotcrete.

Figure 4 presents the effect of varying replacement levels of mineral admixtures on the setting characteristics of the paste. As shown in Figure 4a, the control mixture exhibited initial and final setting times of 4.96 and 10.46 min, respectively. Compared with the control mixture, the incorporation of FA markedly prolonged both the initial and final setting times. When the FA replacement levels were 10%, 20%, and 30%, the initial setting times increased by 14.5%, 46.0%, and 150.6%, respectively, while the final setting times increased more significantly by 76.5%, 157.0%, and 190.9%, respectively. This behavior may be attributed to the spherical morphology of FA particles, which induces a “ball-bearing effect”, which enhances the flowability of the paste and delays the formation of a rigid skeleton structure, thereby prolonging the setting process.

In contrast, the influence of SF (Figure 4b) on the initial setting time was relatively limited, with increases of 2.4%, 13.7%, and 18.5% at replacement levels of 10%, 20%, and 30%, respectively; however, the final setting time decreased by 9.6%, 19.3%, and 30.6%, respectively, indicating that SF accelerated the final setting process. Similarly, MK (Figure 4b) slightly prolonged the initial setting time by 6.0%, 22.6%, and 26.2% at replacement levels of 10%, 20%, and 30%, respectively, whereas the final setting time decreased by 0.4%, 8.6%, and 15.4%, respectively. These results indicate that FA significantly delays the setting process, particularly at higher replacement levels, considering the overall rapid-setting characteristics of the mixtures and the potential experimental deviations associated with timing measurements, the variations in initial setting time induced by SF and MK can be regarded as insignificant. However, at relatively high replacement levels, both SF and MK tended to shorten the final setting time. This may be attributed to their much finer particle size, larger specific surface area, and higher pozzolanic reactivity compared with FA. The fine SF and MK particles can act as nucleation sites for hydration products, accelerating the precipitation and accumulation of C–S–H gels and other hydration products. In addition, MK, owing to its high aluminosilicate reactivity, may further promote the formation of C–A–S–H gels and aluminate-containing hydration products, which contributes to the rapid development of a connected solid skeleton. As a result, although SF and MK may cause only slight changes in the initial setting time, higher dosages can accelerate the later stiffening process and reduce the final setting time.

Figure 4b illustrates how varying mineral admixture contents influence the final setting time of the paste. A trend similar to that observed for the initial setting time is evident for FA, where the final setting time increases with increasing FA content. When the FA replacement levels are 10%, 20%, and 30%, the final setting times are 18.46 min, 26.88 min, and 30.43 min, respectively. This is primarily due to the delayed pozzolanic reaction and the enhanced flowability imparted by FA, both of which retard the setting process. In contrast, the final setting time decreases with increasing contents of SF and MK. For SF, the final setting times at replacement levels of 10%, 20%, and 30% are 9.46 min, 8.44 min, and 7.26 min, respectively. For MK, the corresponding values are 10.42 min, 9.56 min, and 8.85 min. The reduction in final setting time can be attributed to the high pozzolanic reactivity and nucleation effects of SF, which accelerate the hydration process and promote the rapid formation of C–S–H gel. Meanwhile, the flaky morphology and high specific surface area of MK facilitate water adsorption and reduce the free water content in the mixture, thereby decreasing flowability and accelerating the formation of a solid structure.

### 3.2. Workability

#### 3.2.1. Pumpability

**Figure 5:**
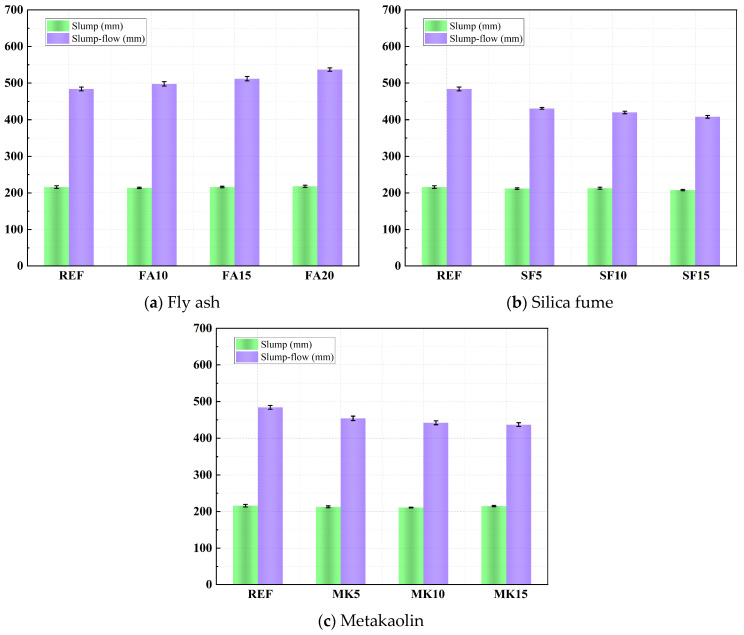
Influence of mineral admixtures on the slump and flow spread of YRS-based shotcrete mixtures.

The workability of Yellow River sediment (YRS)-based shotcrete was evaluated using slump, flow spread, and air content as key performance indices. The effects of mineral admixture type and replacement level on fresh-state behavior were systematically analyzed. The test results indicate that the air content of all mixtures varies within a narrow range of 2.8–3.7%, showing no significant variation among different mix proportions. Therefore, the influence of mineral admixtures on air content is considered negligible and is not discussed further. In contrast, the slump and flow spread are strongly influenced by the type and proportion of mineral admixtures; accordingly, these two parameters are adopted as the primary indicators in the following analysis, as shown in Figure 5.

Figure 5a–c present the influence of various mineral admixtures on the slump and flowability of the mixtures. As indicated in Figure 5a, the addition of fly ash (FA) markedly improves both slump and flow spread characteristics. Moreover, both parameters increase progressively with increasing FA content. Specifically, the slump of mixtures FA10, FA20, and FA30 increases by 5.95%, 11.35%, and 17.84%, respectively, compared with the control mixture, while the corresponding increases in flow spread are 8.26%, 11.30%, and 16.74%. This enhancement is mainly associated with the spherical shape of fly ash (FA) particles, which produces a “ball-bearing” effect. This mechanism reduces internal friction between particles and promotes easier movement within the cementitious system, resulting in improved flowability. In contrast, the incorporation of silica fume (SF) and metakaolin (MK) leads to a reduction in both slump and flow spread, with the magnitude of reduction increasing as the replacement level increases. For SF, the slump decreases by 2.70%, 9.19%, and 18.92% at replacement levels of 5%, 10%, and 15%, respectively, while the corresponding reductions in flow spread are 6.30%, 8.70%, and 17.39%. Similarly, for MK, the slump decreases by 2.70%, 7.03%, and 10.27%, and the flow spread decreases by 1.30%, 3.91%, and 11.52% at replacement levels of 5%, 10%, and 15%, respectively. This behavior is primarily attributed to the extremely high specific surface area and water demand of SF and MK. Their incorporation reduces the thickness of the lubricating water film surrounding solid particles, thereby increasing interparticle friction and flow resistance, which ultimately leads to a decrease in flowability. Overall, the incorporation of FA improves the rheological performance of shotcrete and is beneficial for enhancing pumpability, with the effect becoming more pronounced at higher replacement levels. In contrast, SF and MK reduce flowability and may adversely affect pumpability. Therefore, considering the combined requirements of setting time and workability, the recommended dosage of FA should not exceed 20%, while the dosages of SF and MK should be limited to 15% to ensure adequate fresh-state performance.

#### 3.2.2. Shootability

**Figure 6:**
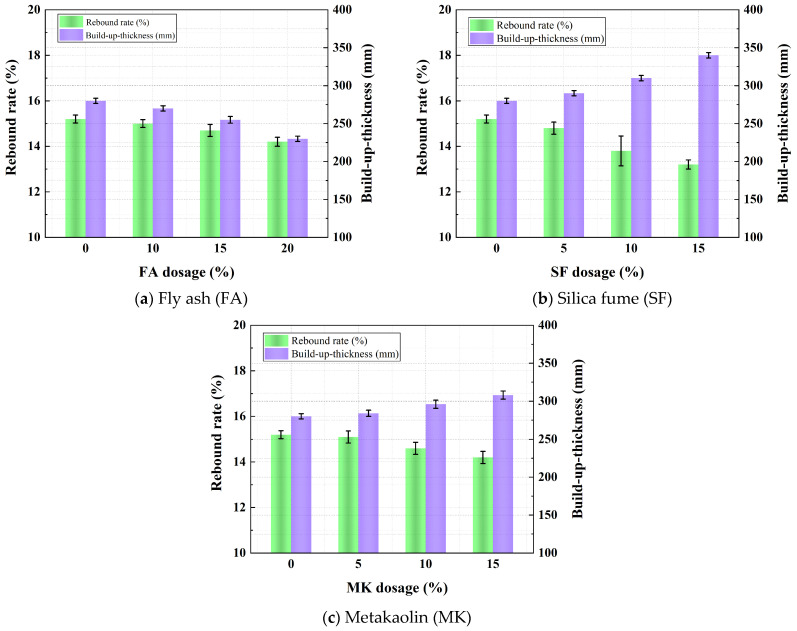
Effects of mineral admixtures on shootability of YRS-based shotcrete.

In this study, fly ash (FA), silica fume (SF), and metakaolin (MK) were used to partially replace cement at different replacement levels. The FA replacement ratios were 10%, 15%, and 20% (denoted as FA10, FA15, and FA20), while SF and MK were incorporated at 5%, 10%, and 15% (denoted as SF5, SF10, SF15, and MK5, MK10, MK15, respectively). The experimental results indicate that different types of mineral admixtures exert significantly different effects on the shootability of shotcrete. Figure 6 illustrates the influence of mineral admixtures on the shootability of YRS-based shotcrete, as evaluated by rebound rate and single-pass build-up thickness. For the control mixture, the rebound rate and build-up thickness were 15.2% and 280 mm, respectively. It can be observed that the incorporation of all three mineral admixtures reduces the rebound rate to varying extents. Specifically, the rebound rate decreases by 1.32%, 3.29%, and 6.58% for FA10, FA15, and FA20, respectively. For SF, the reductions are more pronounced, reaching 2.63%, 9.21%, and 13.16% for SF5, SF10, and SF15, respectively. Similarly, MK reduces the rebound rate by 0.66%, 3.95%, and 6.58% at replacement levels of 5%, 10%, and 15%, respectively. Among the three admixtures, SF exhibits the most significant reduction in rebound rate. In contrast to the rebound behavior, the variation in single-pass build-up thickness shows a different trend. The incorporation of FA leads to a reduction in build-up thickness, which decreases progressively with increasing FA content. Compared with the control mixture, the build-up thickness decreases by 3.57%, 8.93%, and 17.86% for FA10, FA15, and FA20, respectively. This reduction can be attributed to the improved flowability induced by FA, which weakens the thixotropic structural build-up and reduces the ability of the material to adhere and accumulate on the receiving surface. Conversely, the addition of SF and MK enhances the build-up thickness of shotcrete. For SF, the build-up thickness increases by 3.57%, 10.71%, and 21.43% for SF5, SF10, and SF15, respectively, while for MK, the corresponding increases are 1.43%, 5.71%, and 10.00%. This improvement is primarily associated with the high specific surface area and strong water adsorption capacity of SF and MK, which reduce the free water content and enhance the yield stress and structural build-up of the mixture, thereby improving its adhesion and stacking capacity during spraying. Overall, FA exhibits a dual effect on shootability: it reduces rebound rate but simultaneously decreases build-up thickness. In contrast, both SF and MK not only reduce rebound rate but also enhance build-up thickness, thereby improving overall shootability performance. Among the investigated admixtures, SF demonstrates the most pronounced improvement, with the optimal performance observed at a replacement level of 15%.

### 3.3. Strength

#### 3.3.1. Compressive Strength

**Figure 7:**
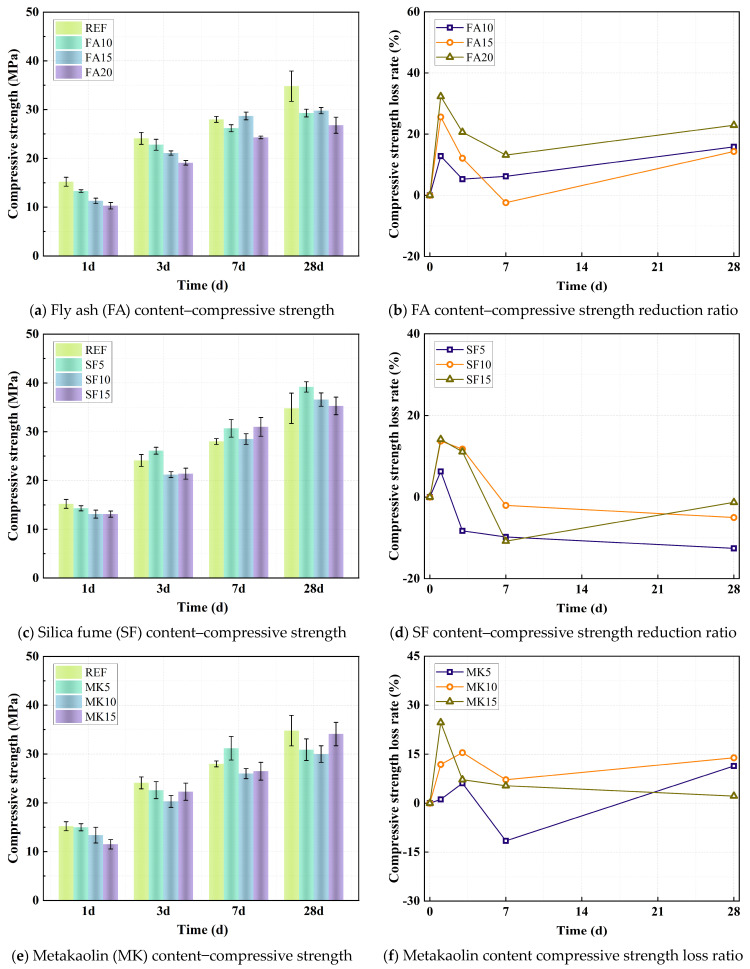
Compressive strength YRS—based shotcrete.

Figure 7 illustrates the effect of mineral admixtures on the compressive strength evolution of Yellow River sediment (YRS)-based shotcrete. Figure 7a,c,e present the variation in compressive strength with different contents of fly ash (FA), silica fume (SF), and metakaolin (MK), respectively, while Figure 7b,d,f depict the corresponding strength reduction ratios calculated from the test data. The control mixture exhibits compressive strengths of 15.2 MPa, 24.1 MPa, and 34.8 MPa at 1 d, 3 d, and 28 d, respectively, indicating a rapid early-age strength development typical of shotcrete systems. The compressive strengths at 1 and 3 days attain 43.68% and 69.13% of the 28-day strength, respectively. Beyond this stage, the strength growth rate slows, reflecting the shift from rapid early-age hydration to a later phase dominated by microstructural densification. The incorporation of different mineral admixtures leads to distinct variations in strength evolution. As shown in Figure 7a, the incorporation of FA results in a significant reduction in compressive strength at all curing ages. At 1 d, the compressive strengths of FA10, FA15, and FA20 are 13.3 MPa, 11.3 MPa, and 10.3 MPa, corresponding to strength reduction ratios of 12.82%, 25.59%, and 32.31%, as shown in Figure 7b. This reduction is primarily attributed to the dilution effect and the relatively low early-age pozzolanic activity of FA, which delays the formation of load-bearing hydration products. With increasing curing age, although the pozzolanic reaction of FA contributes to additional C–S–H formation, the overall strength remains lower than that of the control mixture. At 28 d, the compressive strengths of FA10, FA15, and FA20 are 29.3 MPa, 29.8 MPa, and 26.8 MPa, respectively, indicating that FA exerts a persistent adverse effect on both early- and later-age mechanical performance.

In contrast, SF exhibits a pronounced microfiller and pozzolanic effect, resulting in an age-dependent strength enhancement. As shown in Figure 7c, the early-age (1 d) compressive strength slightly decreases with increasing SF content due to partial cement replacement. However, with continued curing, the strength development rate of SF-modified mixtures surpasses that of the control mixture. At 28 d, the compressive strengths of SF5, SF10, and SF15 reach 39.2 MPa, 36.6 MPa, and 35.3 MPa, respectively, all exceeding the control mixture. This improvement is attributed to the high pozzolanic reactivity, nucleation effect, and particle packing densification of SF, which refine the pore structure, enhance the interfacial transition zone (ITZ), and promote the formation of low Ca/Si ratio C–S–H gel. Among the mixtures, SF5 demonstrates the optimal strengthening effect, indicating that excessive SF may lead to increased water demand and reduced workability, thereby limiting further strength gain. As shown in Figure 7e, MK incorporation results in reduced early-age compressive strength, with 1 d strengths of 15.0 MPa, 13.4 MPa, and 11.5 MPa for MK5, MK10, and MK15, respectively. This behavior is mainly governed by the cement dilution effect and the delayed activation of MK at early ages. However, MK exhibits a pronounced secondary hydration (pozzolanic) reaction at later ages, leading to accelerated strength development. At 7 d, MK5 surpasses the control mixture, indicating enhanced hydration kinetics and microstructural refinement. At 28 d, MK15 achieves a compressive strength of 34.1 MPa, which is comparable to the control mixture. This behavior is attributed to the formation of C–(A)–S–H gel and aluminate phases, which improve matrix densification and pore structure refinement. Overall, the incorporation of mineral admixtures generally reduces early-age compressive strength due to cement dilution and delayed hydration. FA exhibits the most pronounced negative effect, significantly reducing both early- and later-age strength. In contrast, SF provides substantial enhancement in later-age strength due to its combined pozzolanic and microfiller effects, with 5% replacement identified as optimal. MK demonstrates a delayed strengthening mechanism, with significant improvement in later-age strength owing to its high reactivity and contribution to matrix densification. Among the mineral admixtures examined, silica fume (SF) provides the most pronounced improvement in the compressive strength of YRS-based shotcrete.

#### 3.3.2. Splitting Tensile Strength

**Figure 8:**
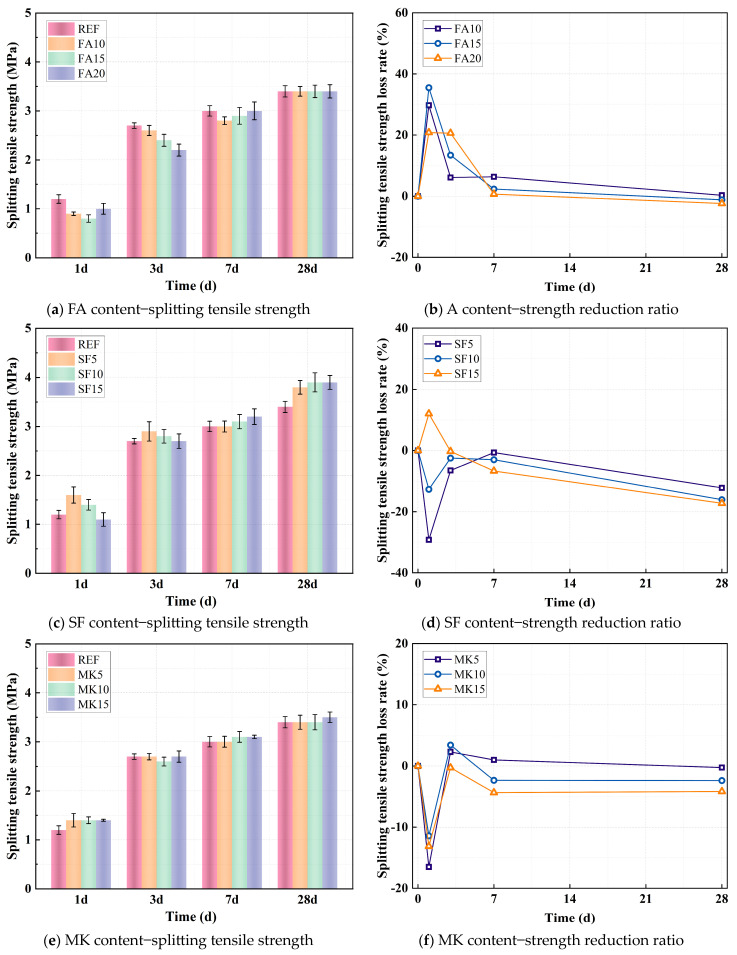
Effects of mineral admixtures on splitting tensile strength of YRS—based shotcrete.

Figure 8 presents the influence of mineral admixtures on the splitting tensile strength of Yellow River sediment (YRS)-based shotcrete. Figure 8a,c,e illustrate the effects of fly ash (FA), silica fume (SF), and metakaolin (MK) contents on splitting tensile strength, while Figure 8b,d,f show the corresponding strength reduction ratios derived from the experimental results. For the control mixture, the splitting tensile strengths at 1 d, 3 d, and 28 d are 1.22 MPa, 2.72 MPa, and 3.36 MPa, respectively. Similar to the compressive strength development, the splitting tensile strength exhibits rapid early-age growth, with 1 d and 3 d strengths reaching 36.31% and 80.95% of the 28 d strength, respectively. The results indicate that different mineral admixtures exert significantly different effects on tensile performance. As shown in Figure 8a, the incorporation of FA reduces the splitting tensile strength at early ages, and the strength generally decreases with increasing FA content. At 1 d, the splitting tensile strengths of FA10, FA15, and FA20 are 0.86 MPa, 0.79 MPa, and 0.97 MPa, corresponding to reductions of 29.75%, 35.51%, and 20.82%, respectively (Figure 8b). This reduction is mainly attributed to the cement dilution effect and the low early-age pozzolanic activity of FA, which delay the formation of a cohesive load-transfer network. However, with increasing curing age, the splitting tensile strength of FA-modified mixtures gradually approaches that of the control. At 28 d, the splitting tensile strengths of FA10, FA15, and FA20 reach 3.35 MPa, 3.40 MPa, and 3.44 MPa, respectively, showing negligible differences compared with the control mixture. This indicates that the later-age pozzolanic reaction of FA contributes to matrix densification and compensates for early strength loss. In contrast, SF significantly enhances the splitting tensile strength at all curing ages. As shown in Figure 8c, at 1 d, the splitting tensile strengths of SF5, SF10, and SF15 are 2.14 MPa, 2.12 MPa, and 2.16 MPa, respectively, all exceeding the control mixture. At 28 d, the strengths further increase to 3.77 MPa, 3.90 MPa, and 3.94 MPa, corresponding to improvements of 12.20%, 16.07%, and 17.26%, respectively (Figure 8d). This enhancement is primarily attributed to the microfiller effect, high pozzolanic reactivity, and ITZ refinement induced by SF, which improve interfacial bonding and crack resistance. The dense C–S–H gel formed by SF effectively bridges microcracks and enhances tensile load transfer capacity.

As shown in Figure 8e, MK also improves the early-age splitting tensile strength. At 1 d, the splitting tensile strengths of MK5, MK10, and MK15 are 1.43 MPa, 1.36 MPa, and 1.38 MPa, respectively, all higher than the control mixture. This improvement is attributed to the high reactivity of MK and its ability to accelerate early hydration and refine the pore structure. With increasing curing age, the strength development rate of MK-modified mixtures becomes comparable to that of the control. At 28 d, the splitting tensile strengths of MK5, MK10, and MK15 are 3.37 MPa, 3.44 MPa, and 3.50 MPa, respectively, indicating comparable or slightly improved tensile performance relative to the control mixture. Overall, FA exhibits a detrimental effect on early-age splitting tensile strength but has a negligible influence at later ages. In contrast, SF significantly enhances splitting tensile strength at both early and later ages, demonstrating the most effective improvement among the three mineral admixtures. MK also contributes positively to early-age tensile strength and maintains comparable performance at later ages. These results indicate that SF is the most effective mineral admixture for improving the tensile performance and crack resistance of YRS-based shotcrete.

#### 3.3.3. Tension–Compression Ratio

**Figure 9:**
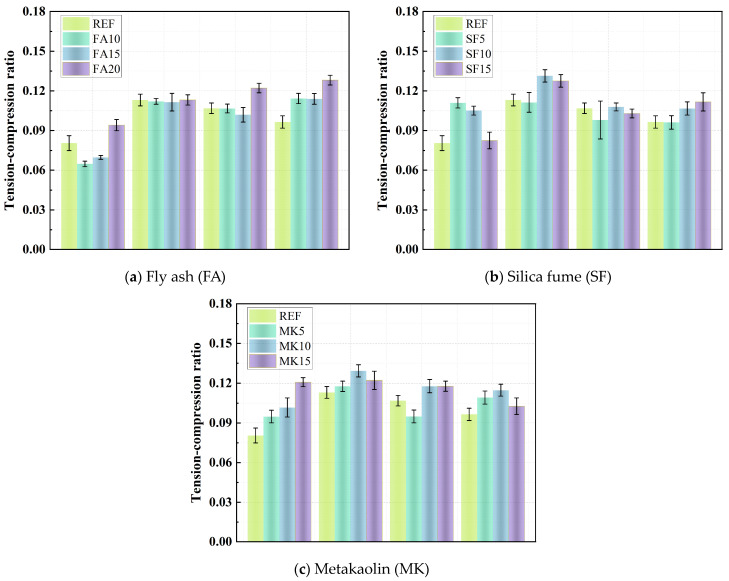
Effects of mineral admixtures on the tension–compression ratio of YRS-based shotcrete.

The variation in the tension–compression ratio (splitting tensile strength to compressive strength) of YRS-based shotcrete under the influence of mineral admixtures is presented in Figure 9. Figure 9a–c illustrate the respective impacts of fly ash (FA), silica fume (SF), and metakaolin (MK) at different replacement levels. As shown in Figure 9, the tension–compression ratio of all mixtures increases progressively with curing age, indicating that the rate of tensile strength development gradually approaches or exceeds that of compressive strength. This trend reflects the continuous refinement of the microstructure and improvement in crack resistance with ongoing hydration. Overall, mixtures incorporating mineral admixtures exhibit higher tension–compression ratios than the control mixture without admixtures. At the same curing age, the tension–compression ratio increases systematically with increasing replacement levels of FA, SF, and MK. However, the underlying mechanisms differ among the three mineral admixtures.

For FA-modified mixtures, the increase in the tension–compression ratio is primarily attributed to the more pronounced reduction in compressive strength compared with splitting tensile strength, which is governed by the cement dilution effect and slower early hydration kinetics. In contrast, SF and MK contribute to an increase in the tension–compression ratio mainly through enhancement of tensile performance. This improvement is associated with pore structure refinement, interfacial transition zone (ITZ) densification, and improved microcrack resistance, which enhance the tensile load transfer capacity of the matrix.

### 3.4. Five-Dimensional Evaluation

**Figure 10:**
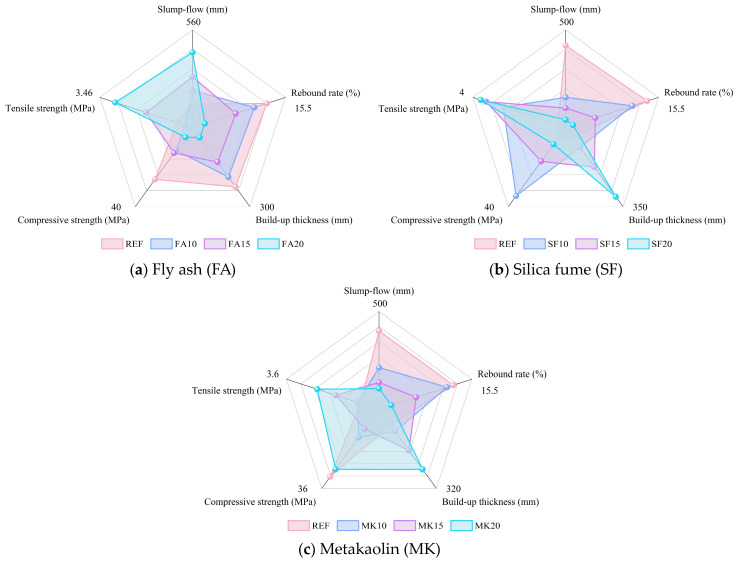
Five-dimensional performance evaluation of Yellow River sediment shotcrete.

Figure 10 illustrates the multi-dimensional performance assessment of YRS-based shotcrete incorporating fly ash (FA), silica fume (SF), and metakaolin (MK). The evaluation integrates five key aspects: setting characteristics, workability, shootability, mechanical properties, and microstructural features. As shown in Figure 10a–c, the performance of shotcrete is significantly influenced by the type of mineral admixture. FA exhibits superior performance in terms of workability and pumpability due to its “ball-bearing effect”, but shows relatively lower mechanical strength and shootability. In contrast, SF demonstrates the most balanced and enhanced overall performance, particularly in shootability and mechanical properties, due to its very fine particle size, high specific surface area, and high amorphous SiO_2_ content. SF can provide additional nucleation sites for cement hydration products and accelerate the pozzolanic consumption of Ca(OH)_2_. This promotes the formation of additional C–S–H gel and improves the compactness of the cementitious matrix. As the SF content increases, this refinement effect becomes more evident; however, which contribute to matrix densification and improved interfacial transition zone (ITZ). MK shows moderate performance across all dimensions, with notable advantages in later-age strength development and microstructural refinement due to the formation of C–(A)–S–H gel. Overall, SF provides the most optimal comprehensive performance, while FA and MK exhibit specific advantages in workability and microstructural enhancement, respectively, highlighting the necessity of selecting appropriate admixtures based on engineering requirements.

## 4. Microstructural Mechanism Analysis

### 4.1. Hydration Product Characteristics

**Figure 11:**
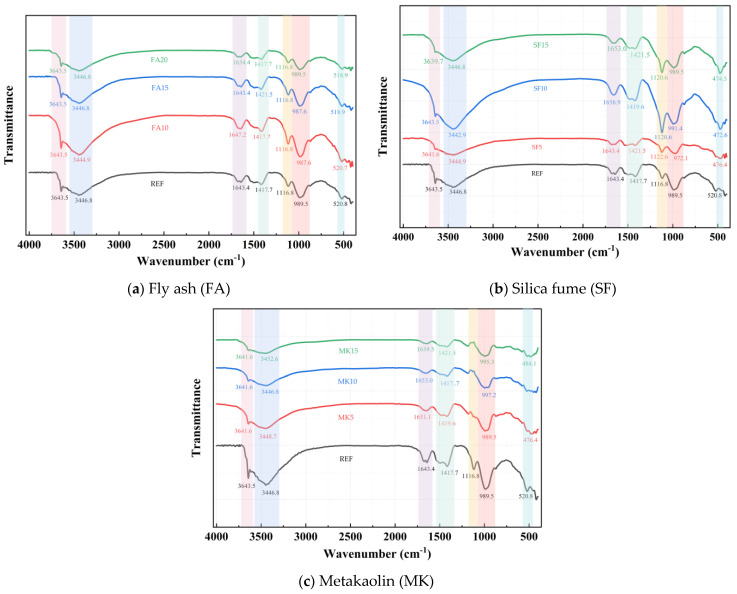
FT–IR spectra of Yellow River sediment (YRS)—based shotcrete with different mineral admixtures.

A combined analysis of X-ray diffraction (XRD), thermogravimetric analysis (TGA), and Fourier transform infrared spectroscopy (FT-IR) was conducted to investigate the effects of multi-source solid wastes and their dosages on the properties of hydration products of YRS-based shotcrete. Figure 11 presents the FT-IR spectra of shotcrete incorporating different solid waste contents. It can be observed that, regardless of whether fly ash (FA), silica fume (SF), or metakaolin (MK) is incorporated, all mixtures exhibit consistent characteristic absorption bands at approximately 3640 cm^−1^ (Ca(OH)_2_), 1100 cm^−1^ (SO_4_^2−^), and 970 cm^−1^ (Si–O stretching vibration). However, noticeable variations in peak intensities are observed among different mixtures.

**Figure 12:**
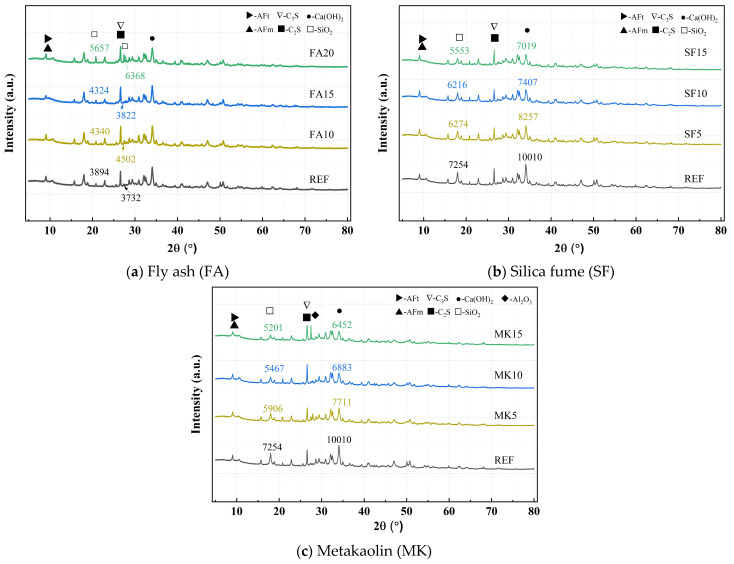
XRD patterns of YRS—based shotcrete incorporating different types and dosages of mineral admixtures.

As shown in Figure 12a, the control mixture (REF) exhibits prominent diffraction peaks corresponding to C_3_S (29.4°) and C_2_S (32.1°), indicating the presence of a substantial amount of unhydrated silicate minerals. With increasing fly ash (FA) content, the intensity of the SiO_2_ peak at 26.58° increases significantly, while the Ca(OH)_2_ (CH) diffraction peak decreases progressively. This clearly demonstrates the consumption of CH through pozzolanic reactions. It is noteworthy that, in the sulfate–aluminate phase region, the diffraction intensity of AFt (ettringite) shows only minor variation, indicating that the formation of sulfate–aluminate hydration products is primarily governed by the type of accelerator, with FA content exerting a relatively limited influence. As illustrated in Figure 12b, with increasing silica fume (SF) content, the diffraction peaks of C_3_S and C_2_S exhibit a systematic reduction, with a particularly pronounced decrease in the C_3_S peak intensity for the SF15 mixture. This phenomenon is mainly associated with the very high specific surface area and pronounced pozzolanic activity of silica fume (SF), which effectively promotes and accelerates the secondary hydration reactions of silicate phases. Meanwhile, the SiO_2_ peak intensity at 26.58° increases linearly with increasing SF content, while the CH peak decreases sharply, indicating that SF markedly enhances CH consumption and promotes the formation of low Ca/Si ratio C–S–H gel. This contributes to improved matrix densification and enhanced mechanical performance. As shown in Figure 12c, increasing metakaolin (MK) content leads to a gradual reduction in both silicate mineral peaks and CH diffraction peaks, indicating that MK promotes the hydration of silicate phases. Although the AFt peak intensity exhibits limited variation, the emergence of additional diffraction peaks adjacent to the AFt region suggests the formation of carboaluminate phases. This is attributed to the high Al_2_O_3_ content of MK, which participates in secondary hydration reactions and facilitates the generation of AFm-type phases, thereby enhancing the compactness of the matrix.

**Figure 13:**
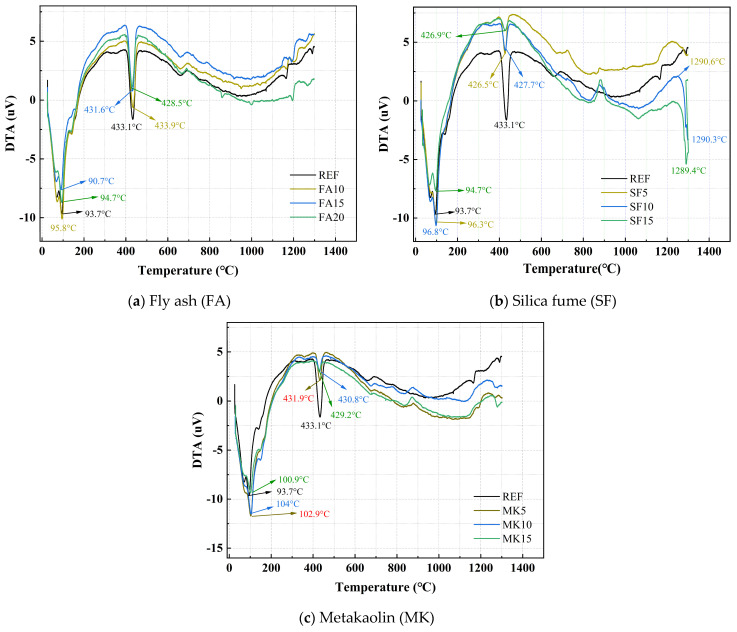
TGA/DTG curves of YRS—based shotcrete with different mineral admixtures.

Figure 13 presents the thermogravimetric (TGA) results of YRS-based shotcrete incorporating different types and dosages of mineral admixtures. As observed, three characteristic mass-loss regions can be identified: 80–100 °C (decomposition of AFt), 110–120 °C (dehydration of C–S–H gel), and 440–450 °C (decomposition of Ca(OH)_2_ (CH)). The mass-loss behavior in these temperature ranges exhibits systematic variations depending on the type and dosage of mineral admixtures. As shown in Figure 13a, no new thermal decomposition peaks are observed with increasing fly ash (FA) content, indicating that the incorporation of FA does not alter the types of hydration products formed in the system. However, the intensities of the characteristic peaks vary significantly. In the 80–120 °C range (associated with AFt and AFm decomposition), the FA15 mixture exhibits a noticeably lower peak intensity compared with the control group (REF). Similarly, in the 440–450 °C range (CH decomposition), a significant reduction in peak intensity is observed. These results indicate that: (i) the reactive SiO_2_ and Al_2_O_3_ in FA consume CH through pozzolanic reactions, forming additional C–(A)–S–H gel; and (ii) FA exerts a dual effect on sulfate–aluminate phases—at low dosages (<10%), the supply of Al promotes AFt formation, whereas at higher dosages, the dilution effect suppresses the formation of these phases. Furthermore, the relatively stable mass loss in the 600–800 °C range (CaCO_3_ decomposition) suggests that FA content has a limited influence on carbonation behavior. These findings are consistent with the XRD results.

As illustrated in Figure 13b, with increasing silica fume (SF) content, the decomposition peaks associated with AFt and AFm phases first increase and then decline, while the peak corresponding to calcium hydroxide (CH) shows a pronounced reduction in intensity. This trend is in good agreement with the XRD analysis, confirming that SF markedly accelerates CH consumption and promotes the hydration of C_3_S and C_2_S phases. Notably, when the SF content exceeds 10%, two additional endothermic peaks appear at approximately 800–900 °C and 1200–1300 °C. The former is attributed to the phase transformation of amorphous SiO_2_ (e.g., α-quartz to β-quartz transition), while the latter may correspond to the formation of calcium aluminosilicate phases. These high-temperature phase transformations may result from: (i) the agglomeration of unreacted nano-SiO_2_ at high dosages; and (ii) solid-state reactions between free SiO_2_ and aluminate hydration products (e.g., C_3_A-derived phases).

As shown in Figure 13c, the incorporation of metakaolin (MK) produces a trend similar to that of SF. The CH-related mass-loss peak decreases significantly with increasing MK content, confirming its strong pozzolanic reactivity, which promotes CH consumption. In the 80–120 °C range, the mass loss initially increases and then decreases, with the MK5 mixture showing the highest value, while the MK15 mixture returns to a level comparable to the control. This trend is consistent with the variation in AFt diffraction peak intensity observed in the XRD results. In the 440–450 °C range, the continuous reduction in CH-related peak intensity further confirms the strong reactivity of Al_2_O_3_ and SiO_2_ in MK. Additionally, a new mass-loss peak appears in the 600–650 °C range, which is attributed to the decomposition of carboaluminate phases (AFm–CO_3_). This indicates that Al_2_O_3_ in MK participates in sulfate–aluminate reactions, leading to the formation of additional hydration products and contributing to matrix densification.

### 4.2. Matrix Microstructure

#### 4.2.1. Effect of FA Content on Matrix Microstructure

**Figure 14:**
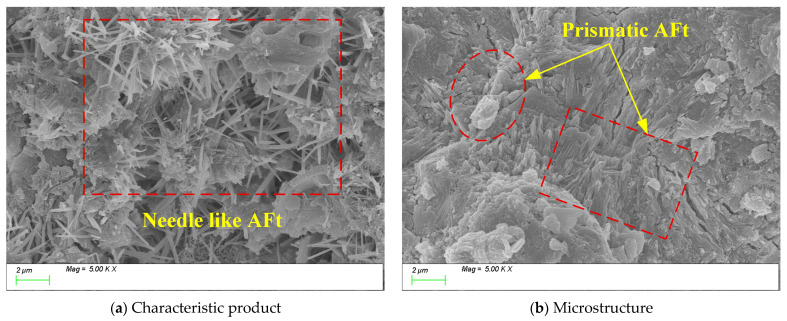
SEM images of the control mixture (REF) at 28 days.

Figure 14a,b present the microstructural morphology of the control mixture (REF) after 28 d of hydration. As shown in Figure 14a, a large number of prismatic AFt (ettringite) crystals can be clearly observed within the matrix. This morphology is typical for shotcrete systems incorporating alkali-free accelerators. The formation mechanism can be attributed to the addition of the accelerator, which not only significantly accelerates the hydration of C_3_A phases in cement but also continuously supplies Al^3+^ and SO_4_^2−^ ions to the pore solution. This promotes favorable conditions for the nucleation and growth of AFt. Unlike the slender needle-like AFt crystals commonly observed in conventional cementitious systems, the AFt in this study predominantly exhibits prismatic and short rod-like morphologies. This morphological variation reflects the pronounced influence of the accelerator on the crystallization behavior of hydration products. In addition to the morphological transformation, the quantity of AFt is also significantly increased. As illustrated in Figure 14b, after 28 d of hydration, the matrix contains not only abundant prismatic AFt crystals but also a substantial amount of dense and continuous flocculent C–S–H gel. The C–S–H gel exhibits a typical layered or interwoven structure, forming an interconnected network with hexagonal plate-like Ca(OH)_2_ (CH) and prismatic AFt crystals. This compact and well-integrated microstructure significantly enhances the internal bonding and reduces pore connectivity. Such a dense microstructural configuration is considered one of the primary reasons for the rapid development of early-age strength and the stable gain of long-term mechanical properties.

**Figure 15:**
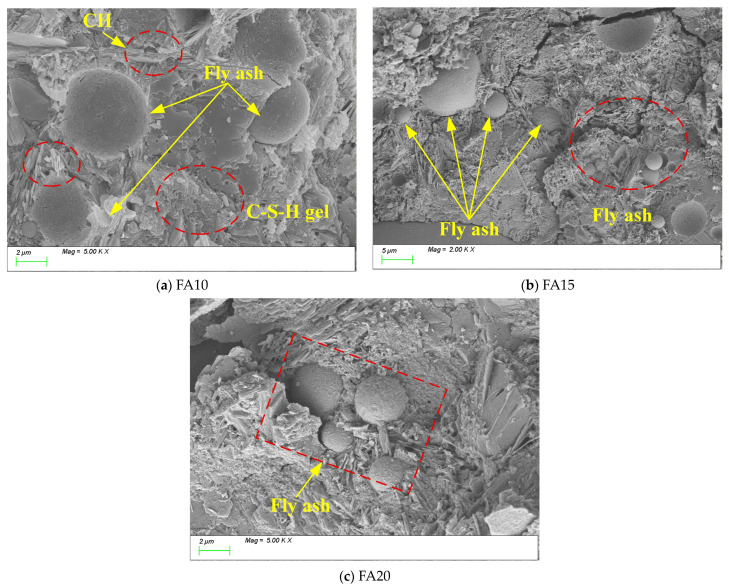
Effect of FA content on matrix microstructure.

As shown in Figure 15a, corresponding to the FA10 mixture, a relatively dense matrix structure can be observed. A limited number of unreacted fly ash (FA) particles are present; however, their surfaces are covered with a substantial amount of coral-like C–S–H gel formed through pozzolanic reactions. Additionally, flower-like C–S–H gel structures are distributed within the matrix. This morphology can be attributed to the secondary reaction between the active SiO_2_ in FA and Ca(OH)_2_ generated during cement hydration, which modifies the nucleation and growth mechanism of hydration products. These observations indicate that FA not only contributes to matrix densification through a filler effect but also enhances the microstructure via pozzolanic activity. A similar microstructural pattern is observed in Figure 15b for the FA15 mixture. Although some unreacted FA particles remain, the matrix maintains a relatively compact structure. The surfaces of FA particles are coated with coral-like C–S–H gel, while flower-like C–S–H formations are also evident within the matrix. Compared with FA10, the extent of pozzolanic reaction appears more pronounced; however, the overall distribution of hydration products remains relatively uniform, contributing to acceptable microstructural integrity. In contrast, the microstructure of the FA20 mixture, shown in Figure 14c, exhibits several distinct characteristics. A higher value of unreacted FA particles is clearly visible, with a more heterogeneous particle size distribution. Although coral-like C–S–H gel still forms on the surfaces of FA particles and flower-like C–S–H gel is present within the matrix, the overall uniformity of hydration products is reduced. More importantly, microstructural defects such as microcracks can be observed. These phenomena are primarily attributed to the excessive FA content, which leads to insufficient availability of Ca(OH)_2_ for pozzolanic reactions and a pronounced dilution effect. Consequently, the continuity and compactness of the cementitious matrix are weakened, adversely affecting both microstructural integrity and mechanical performance.

#### 4.2.2. Effect of SF Content on Matrix Microstructure

**Figure 16:**
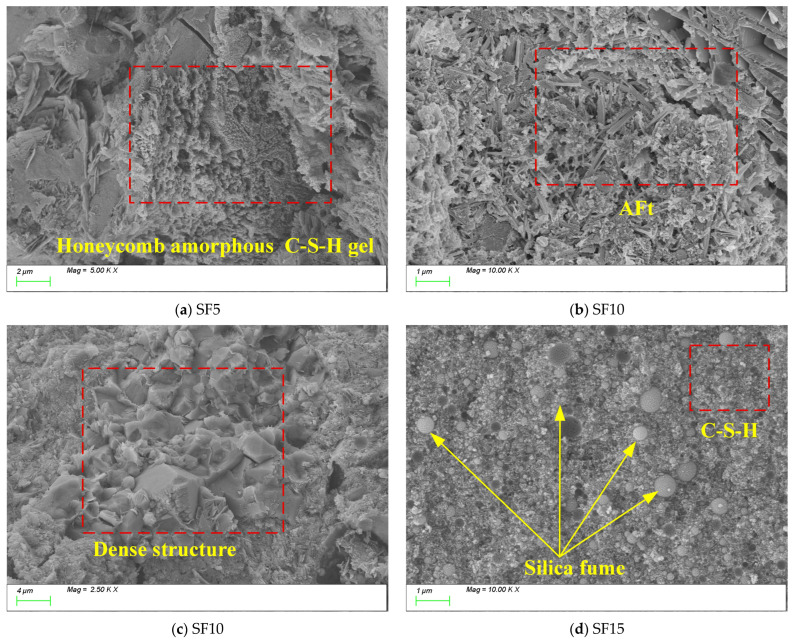
Effect of SF content on matrix microstructure.

As shown in Figure 16a (SF5), a considerable amount of plate-like hydration products and C–S–H gel can be observed within the matrix. The C–S–H gel exhibits a typical honeycomb-like morphology, indicating a relatively low degree of polymerization and weak interparticle bonding. This can be attributed to the limited dosage of silica fume (5%), which results in an insufficient pozzolanic reaction despite the presence of reactive amorphous SiO_2_. Consequently, the matrix remains relatively loose, which is not favorable for strength development. In Figure 16b (SF10), the microstructure becomes more complex, characterized by the coexistence of rod-like AFt (ettringite), C–S–H gel, and large plate-like crystalline products. These hydration products form an interconnected three-dimensional network, where AFt crystals and C–S–H gel are interwoven. However, noticeable microcracks are observed at the interfaces between large plate-like products and the surrounding matrix. This behavior may be attributed to: (i) elastic modulus mismatch between the plate-like phases and the matrix leading to stress concentration; (ii) crystallization pressure induced by rapid AFt formation; and (iii) incomplete consumption of Ca(OH)_2_ due to the non-optimal SF dosage (10%). Although the matrix compactness is improved compared with SF5, the deformation compatibility between hydration products remains limited. As illustrated in Figure 16c, the matrix exhibits a highly dense microstructure, with C–S–H gel forming a compact, block-like morphology. This is mainly due to the extremely fine particle size and high specific surface area of silica fume, which significantly enhances its reactivity. The amorphous SiO_2_ in SF reacts rapidly with Ca(OH)_2_, producing additional C–S–H gel with a low Ca/Si ratio. This type of C–S–H gel possesses a higher degree of polymerization and superior mechanical properties, resulting in a denser packing structure and a refined interfacial transition zone (ITZ). This microstructural refinement is a key factor contributing to the enhanced strength performance.

In contrast, Figure 16d (SF15) indicates that a portion of silica fume remains unreacted, maintaining its original spherical or near-spherical shape with relatively smooth surfaces. These particles are tightly encapsulated by dense C–S–H gel, forming a characteristic “micro-aggregate–gel” composite structure. Compared with lower SF dosages, the SF15 mixture exhibits a significantly more compact and homogeneous matrix. This pronounced densification can be attributed to three synergistic mechanisms: (i) the micro-filling effect of ultrafine SF particles, which reduces porosity and refines the pore structure; (ii) strong mechanical interlocking and chemical bonding between SF particles and C–S–H gel; and (iii) the sufficient availability of reactive SiO_2_ at higher SF content, which promotes extensive pozzolanic reactions and the formation of large amounts of low Ca/Si ratio C–S–H gel. These combined effects substantially enhance the microstructural integrity and overall mechanical performance of the matrix.

#### 4.2.3. Effect of MK Content on Matrix Microstructure

**Figure 17:**
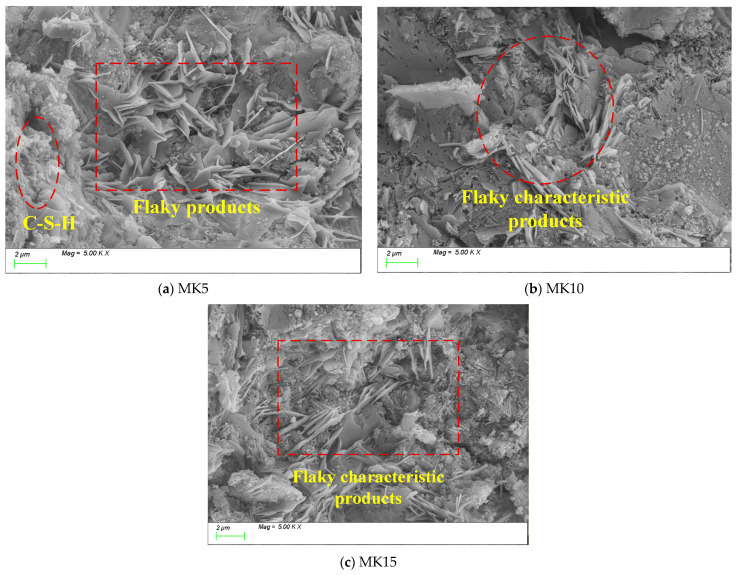
Effect of MK content on matrix microstructure.

As shown in Figure 17a (MK5), the incorporation of 5% metakaolin results in the formation of distinct hydration products within the matrix. Two types of plate-like phases can be clearly identified: (i) hexagonal plate-like Ca(OH)_2_ (CH) crystals with sizes ranging from 5–10 μm, representing typical cement hydration products; and (ii) larger, well-defined plate-like particles with smooth surfaces and sharp edges, which are identified via EDS analysis as unreacted metakaolin with an Al/Si ratio close to 1:1. In addition, a considerable number of rod-like AFt (ettringite) crystals with lengths of 2–5 μm are observed, exhibiting prismatic morphology and interweaving with C–S–H gel. At this dosage, the C–S–H gel predominantly retains coral-like (high specific surface area) and flower-like (layered stacking) morphologies, indicating that 5% MK is insufficient to significantly modify the fundamental formation mechanism of hydration products. Notably, interfacial defects are evident, particularly around large unreacted MK particles, where loose zones and microcrack networks with widths of approximately 0.5–2 μm are observed, which are detrimental to matrix compactness. In Figure 17b (MK10), although the types of hydration products remain similar (including CH crystals, AFt, and residual MK particles), the microstructure exhibits significant refinement compared with MK5. The pozzolanic activity of C–(A)–S–H gel increases markedly, which can be attributed to the higher MK content. The enhanced pozzolanic activity promotes the consumption of CH through reactions involving active Al_2_O_3_ and SiO_2_, while the incorporation of Al into the C–S–H structure leads to the formation of C–(A)–S–H gel with a reduced Ca/(Si + Al) ratio. This results in a higher degree of polymerization and improved structural integrity. Furthermore, the size of unreacted MK particles decreases, indicating a more complete reaction. The C–(A)–S–H gel exhibits a denser, wrinkled morphology, providing stronger mechanical interlocking compared with the coral-like structure observed in MK5. These microstructural enhancements contribute directly to improved mechanical performance. As illustrated in Figure 17c (MK15), the hydration product types remain generally consistent with those observed in MK5 and MK10; however, the microstructure shows several notable evolutions. First, the spatial distribution of CH crystals and residual MK particles transitions from a dispersed arrangement to a more compact, clustered configuration, forming a more continuous and interconnected matrix skeleton. Second, the content of C–(A)–S–H gel further increases compared with MK10, indicating a higher degree of pozzolanic reaction and greater incorporation of Al into the gel structure. This contributes significantly to matrix densification and pore refinement. Moreover, the enhanced gel connectivity improves load transfer efficiency within the matrix. However, at higher MK dosage, potential agglomeration effects and local heterogeneity may still occur, which could influence uniformity if not properly dispersed.

## 5. Conclusions

This study investigated the effects of fly ash (FA), silica fume (SF), and metakaolin (MK) on the fresh, mechanical, and microstructural properties of Yellow River sediment (YRS)-based shotcrete. The main conclusions are summarized as follows:

(1) The type and dosage of mineral admixtures significantly affect the setting behavior of YRS-based shotcrete. FA, with low pozzolanic reactivity and a “ball-bearing effect,” prolongs both initial and final setting times as dosage increases. SF accelerates hydration, while MK shortens the final setting time due to its plate-like morphology and water adsorption. Both SF and MK have a limited effect on the initial setting time.
(2) FA improves slump and flow spread, enhancing pumpability, but reduces build-up thickness during spraying. SF and MK reduce flowability, due to high surface area and water demand, but lower rebound and increase build-up thickness. SF shows the greatest improvement in shootability, with a 21.4% increase at 15% dosage. Recommended dosages are ≤20% for FA and ≤15% for SF and MK.
(3) FA consistently reduces compressive strength, though its effect on splitting tensile strength decreases over time, with 28-day values close to the reference. SF enhances later-age strength through pozzolanic reactions; at 5% dosage, compressive strength exceeds the reference after 3 days and reaches 39.2 MPa at 28 days, with improved splitting tensile strength. MK shows delayed strength development, achieving 28-day compressive strength comparable to the reference at 15% dosage, while improving early-age splitting tensile strength.
(4) Microstructural analyses revealed that SF and MK promoted the consumption of Ca(OH)_2_ and the formation of additional C–(A)–S–H/C–S–H gels, resulting in a denser matrix and improved interfacial transition zone. Excessive FA led to unreacted particles and microcracks.
(5) From a microstructural perspective, FA promotes the formation of C–(A)–S–H gel through pozzolanic reactions, although excessive FA results in unreacted particles and microcracks. SF enhances the formation of low Ca/Si ratio C–S–H gel, producing a denser matrix and a refined interfacial transition zone (ITZ).
(6) Based on the experimental results obtained in this study, the mixture incorporating YRS with 10% silica fume (SF10) exhibited the most balanced overall performance among the investigated mixtures. This superior performance can be attributed to its relatively rapid setting behavior, enhanced mechanical strength, improved shootability, and denser microstructure. However, this recommendation is derived from laboratory-scale observations under the specific experimental conditions of the present study. Since long-term durability, field spraying performance, and practical construction adaptability were not evaluated, further durability assessments and field-scale validation are still required before this mixture can be confidently recommended for practical engineering applications.
(7) Compared with these conventional mineral admixtures, Yellow River sediment is characterized by finer particles and a relatively high clay content. These characteristics may reduce the workability, strength development, and overall performance of shotcrete to some extent. Therefore, although the utilization of Yellow River sediment provides a feasible approach for resource recycling and sustainable shotcrete production, further performance enhancement is still required. Accordingly, the development of high-performance Yellow River sediment-based shotcrete will be an important direction for future research.

## Figures and Tables

**Table 1:** Chemical compositions of YRS and mineral admixtures (wt.%) [29].

Minerals	SiO_2_	Al_2_O_3_	Fe_2_O_3_	K_2_O	TiO_2_	MgO	CaO	Other
YRS	68.64	12.33	3.25	2.55	0.74	2.05	8.40	2.04
Cement	22.64	4.68	3.57	—	—	2.94	64.88	1.29
FA	45.89	20.25	16.45	4.27	2.41	0.22	7.24	3.34
SF	98.87	0.96	0.07	0.78	—	0.36	0.41	0.75
MK	53.52	44.52	0.92	0.74	0.18	0.07	—	0.07

**Table 2:** Mixture proportions for setting time test.

No.	Cement	CP	FA	SF	MK	Accelerator	Water	SP
REF	0.685	—	—	—	—	0.055	0.260	—
FA10	0.548	0.068	0.068	—	—	0.055	0.260	—
FA20	0.479	0.068	0.137	—	—	0.055	0.260	—
FA30	0.411	0.068	0.205	—	—	0.055	0.260	—
SF10	0.541	0.068	—	0.068	—	0.054	0.257	0.014
SF20	0.473	0.068	—	0.135	—	0.054	0.257	0.014
SF30	0.405	0.068	—	0.203	—	0.054	0.257	0.014
MK10	0.544	0.068	—	—	0.068	0.054	0.259	0.007
MK20	0.476	0.068	—	—	0.136	0.054	0.259	0.007
MK30	0.408	0.068	—	—	0.204	0.054	0.259	0.007

**Table 3:** Mixture proportions for workability and mechanical strength tests.

No.	YRS	Sand	Cement	FA	SF	MK	Accelerator	Water	SP
REF	0.398	0.100	0.341	—	—	—	0.027	0.127	0.007
FA10	0.398	0.100	0.307	0.034	—	—	0.027	0.127	0.007
FA15	0.398	0.100	0.290	0.051	—	—	0.027	0.127	0.007
FA20	0.398	0.100	0.273	0.068	—	—	0.027	0.127	0.007
SF5	0.397	0.099	0.323	—	0.017	—	0.027	0.127	0.011
SF10	0.397	0.099	0.306	—	0.034	—	0.027	0.127	0.011
SF15	0.397	0.099	0.289	—	0.051	—	0.027	0.127	0.011
MK5	0.397	0.099	0.323	—	—	0.017	0.027	0.127	0.011
MK10	0.397	0.099	0.306	—	—	0.034	0.027	0.127	0.011
MK15	0.397	0.099	0.289	—	—	0.051	0.027	0.127	0.011

**Table 4:** Mixture proportions for microstructural tests.

No.	Cement	CP	FA	SF	MK	Accelerator	Water	SP
REF	0.685	—	—	—	—	0.055	0.260	—
FA10	0.548	0.068	0.068	—	—	0.055	0.260	—
FA15	0.514	0.068	0.103	—	—	0.055	0.260	—
FA20	0.479	0.068	0.137	—	—	0.055	0.260	—
SF5	0.574	0.068	—	0.034	—	0.054	0.257	0.014
SF10	0.541	0.068	—	0.068	—	0.054	0.257	0.014
SF15	0.507	0.068	—	0.101	—	0.054	0.257	0.014
MK5	0.578	0.068	—	—	0.034	0.054	0.259	0.007
MK10	0.544	0.068	—	—	0.068	0.054	0.259	0.007
MK15	0.510	0.068	—	—	0.102	0.054	0.259	0.007

Note: FA, MK, and SF represent fly ash, metakaolin, and silica fume, respectively. CP stands for clay powder, SP stands for superplasticizer, FA10 indicates a fly ash content of 10%, and so on for the others.

**Table 5:** Classification of tests into workability, mechanical properties, hydration kinetics, and microstructural characterization.

Properties	Performance Index	Specimen Size	Quantity
Setting time	initial setting time	—	30
	final setting time	—	30
Pumpability	slump	—	30
	slump flow	—	30
Shootability	rebound rate	—	30
	Build-up-thickness	—	30
Strength	compressive strength		
	splitting tensile strength	100 mm	240
Characteristic products	TGA	40 mm	30
	XRD	40 mm	30
	Infrared spectroscopy analysis	40 mm	30
Microstructural properties	scanning electron microscopy	40 mm × 40 mm × 40 mm	30

## Data Availability

The original contributions presented in this study are included in the article. Further inquiries can be directed to the corresponding author.
